# The 2018 Lebanese Society of Infectious Diseases and Clinical Microbiology Guidelines for the use of antimicrobial therapy in complicated intra-abdominal infections in the era of antimicrobial resistance

**DOI:** 10.1186/s12879-019-3829-2

**Published:** 2019-03-29

**Authors:** Nicholas Haddad, Souha S. Kanj, Lyn S. Awad, Dania I. Abdallah, Rima A. Moghnieh

**Affiliations:** 1 Internal Medicine-Infectious Disease, Central Michigan University, Saginaw, MI 48602 USA; 2 Division of Infectious Diseases, Department of Internal Medicine, American University of Beirut Medical Center, Beirut, Lebanon; 3 Pharmacy Department, Makassed General Hospital, Beirut, Lebanon; 4 Pharmacy Department, Makassed General Hospital, Beirut, Lebanon; 5 Division of Infectious Diseases, Department of Internal Medicine, Makassed General Hospital, Beirut, Lebanon

**Keywords:** Antimicrobial resistance, Antimicrobial stewardship, Antimicrobial therapy, Complicated intra-abdominal infections, Guidelines, Lebanon

## Abstract

**Background:**

The Lebanese Society of Infectious Diseases and Clinical Microbiology (LSIDCM) is involved in antimicrobial stewardship. In an attempt at guiding clinicians across Lebanon in regards to the proper use of antimicrobial agents, members of this society are in the process of preparing national guidelines for common infectious diseases, among which are the guidelines for empiric and targeted antimicrobial therapy of complicated intra-abdominal infections (cIAI). The aims of these guidelines are optimizing patient care based on evidence-based literature and local antimicrobial susceptibility data, together with limiting the inappropriate use of antimicrobials thus decreasing the emergence of antimicrobial resistance (AMR) and curtailing on other adverse outcomes.

**Methods:**

Recommendations in these guidelines are adapted from other international guidelines but modeled based on locally derived susceptibility data and on the availability of pharmaceutical and other resources.

**Results:**

These guidelines propose antimicrobial therapy of cIAI in adults based on risk factors, site of acquisition of infection, and clinical severity of illness. We recommend using antibiotic therapy targeting third-generation cephalosporin (3GC)-resistant gram negative organisms, with carbapenem sparing as much as possible, for community-acquired infections when the following risk factors exist: prior (within 90 days) exposure to antibiotics, immunocompromised state, recent history of hospitalization or of surgery and invasive procedure all within the preceding 90 days. We also recommend antimicrobial de-escalation strategy after culture results. Prompt and adequate antimicrobial therapy for cIAI reduces morbidity and mortality; however, the duration of therapy should be limited to no more than 4 days when adequate source control is achieved and the patient is clinically stable. The management of acute pancreatitis is conservative, with a role for antibiotic therapy only in specific situations and after microbiological diagnosis. The use of broad-spectrum antimicrobial agents including systemic antifungals and newly approved antibiotics is preferably restricted to infectious diseases specialists.

**Conclusion:**

These guidelines represent a major step towards initiating a Lebanese national antimicrobial stewardship program. The LSIDCM emphasizes on development of a national AMR surveillance network, in addition to a national antibiogram for cIAI stratified based on the setting (community, hospital, unit-based) that should be frequently updated.

## Background

Complicated intra-abdominal infections (cIAI) remain a major challenge in clinical practice. They are the main cause of postoperative morbidity following abdominal surgery and the most frequent cause for admission to a surgical intensive care unit [1, 2]. Intra-abdominal infections (IAI) represent diverse disease processes requiring different approaches for their management; the disease spectrum ranges from simple uncomplicated IAIs to severe infections with septic shock [2, 3].

The management of cIAI relies on 3 major pillars: surgical intervention to achieve source control, fluid and electrolyte resuscitation especially in sick patients, and sepsis management including antimicrobial therapy. Early diagnosis and prompt initiation of adequate antimicrobial therapy help in improving patient outcomes [3, 4]. Guidelines for antimicrobial management have been published by several international societies. Although causative organisms for cIAI are almost the same across countries and continents, their antimicrobial susceptibility patterns vary widely between geographic regions, and even among different institutions within the same country [3]. With that in mind and based on principles of antimicrobial stewardship [5], the judicious use of antimicrobials is mandatory, with the urgent need to establish national guidelines that tailor antibiotic choices based on local susceptibility data in each country [6]. Consequently, the availability of national guidelines for appropriate management of cIAI becomes a priority.

The LSIDCM is a specialists’ society, which is part of the Lebanese Order of Physicians, whose members are registered infectious diseases physicians from different educational and training backgrounds, along with registered clinical microbiologists. The LSIDCM has published guidelines for the management of common infections in adults including community acquired pneumonia [7], urinary tract infections [8], and febrile neutropenia [9], and is in the process of preparing national guidelines for other types of infections. A subgroup of the society members was tasked with drafting the guidelines. We herein propose guidelines for the antimicrobial management of cIAI.

In view of increasing antimicrobial resistance, treating patients with cIAIs has become more challenging, globally [3, 10] and nationally [11]. Rising resistance to 3rd generation cephalosporins (3GC) and fluoroquinolones [10, 12], along with emerging carbapenem resistance in Gram-negative bacteria have become widespread [13]. Delaying adequate antibiotic therapy significantly increases the risk of mortality [14]. Moreover, the universal use of broad-spectrum antibiotics leads to collateral damage including emergence of *Clostridium difficile* infections and development of antimicrobial resistance [15, 16]. This has been the driving force behind the publication of this set of guidelines. Based on the review of international guidelines and local antimicrobial susceptibility profiles, the LSIDCM aims to formulate recommendations consistent with the Lebanese susceptibility data, considering the availability of certain antimicrobial agents in the Lebanese pharmaceutical market or lack thereof, in an attempt to streamline clinical practice across the nation. The ultimate goals are optimizing patient care according to evidence-based medicine, while utilizing principles of antimicrobial stewardship.

## Methods

We reviewed the epidemiology of antimicrobial resistance in Lebanon. Our recommendations are adapted from other international guidelines but modeled based on locally derived susceptibility data and on the availability of pharmaceutical and other resources. Recommendations are restricted to the antimicrobial management of cIAI in adults and these guidelines will not provide detailed recommendations about diagnosis and surgical interventions of these infections.

The cIAI (biliary and extra-biliary) guidelines reviewed herein are:

- The 2010 Surgical Infection Society and Infectious Diseases Society of America consensus guidelines for the diagnosis and management of complicated intra-abdominal infection in adults and children [1].
- The 2010 Canadian practice guidelines for surgical intra-abdominal infections [4].
- The 2014 Asian Consensus Taskforce on Complicated Intra-abdominal Infections guidelines for antibiotic management of complicated intra-abdominal infections in adults [17].
- The 2015 French Society of Anesthesia and Intensive Care guidelines for the management of intra-abdominal [18].
- The 2017 Surgical Infection Society revised guidelines for the management of intra-abdominal infection [19].
- The 2017 World Society of Emergency Surgery guidelines for the management of intra-abdominal infections [3].
- The 2018 Tokyo guidelines for the antimicrobial therapy in acute cholangitis and cholecystitis [20].

The acute pancreatitis guidelines reviewed herein are:

- The 2013 American College of Gastroenterology guidelines for the management of acute pancreatitis [21].
- The 2013 International Association of Pancreatology/American Pancreatic Association working group guidelines for the management of acute pancreatitis [22].
- The 2015 Japanese guidelines for the management of acute pancreatitis [23].

### Level of Evidence

The level of evidence of the recommendations follows the LSIDCM grading [8, 9]. We adopted this evidence grading from the National Comprehensive Cancer Network guidelines for febrile neutropenia [24]. (Table 1).

**Table 1:** Grading system for the level of evidence of recommendations adopted by the Lebanese Society of Infectious Diseases and Clinical Microbiology (LSIDCM)

Grading category	Definition
1	Based upon high-level evidence with multiple well-designed, controlled, randomized blinded studies and meta-analysis. There is uniform consensus that the intervention is adequate.
2A	Based upon lower level of well-controlled, non- blinded or randomized studies, with retrospective reviews. There is uniform consensus that the intervention is adequate.
2B	Based upon lower level of well-controlled, non- blinded or randomized studies, with retrospective reviews. There is majority consensus that the intervention is adequate.
3A	Based upon any evidence that is less than well-controlled, or randomized, or large sample studies, mostly retrospective. There is uniform consensus that the intervention is adequate.
3B	Based upon any evidence that is less than well-controlled, or randomized, or large sample studies, mostly retrospective. There is no uniform consensus that the intervention is adequate.
3C	Based upon any evidence that is less than well-controlled, or randomized, or large sample studies, mostly retrospective. There is no consensus that the intervention is adequate.
4A	There is any level of evidence from literature against the intervention. There is uniform consensus against the intervention.

## Results

### Microbiological data: Antimicrobial susceptibility in Lebanon

The only available antibiotic susceptibility data from Lebanon that describe IAI are derived from the Study for Monitoring Antimicrobial Resistance Trends (SMART), in which prospective data were collected from IAI in different medical centers across Jordan and Lebanon between 2011 and 2013 [25]. The percentage of resistance to third generation cephalosporins (forwardly referred to as 3GC) in Enterobacteriaceae in this study was 31.5% [25]. However, this proportion comes from pooled data from Lebanon and Jordan together [25]. The specimens from Lebanon were collected from 2 university hospitals [25]. The recovered organisms were not stratified into community-acquired or nosocomial [25]. These caveats of SMART prevented us from using its results as absolute epidemiologic background for this set of guidelines.

Nevertheless, the microbiology of cIAI is well described in the literature. It consists mainly of Enterobacteriaceae, with *Escherichia coli* being the leading organism, followed by *Klebsiella spp*, other Enterobacteriaceae, *Pseudomonas spp*, *Enterococci*, anaerobes and *Candida* species [1, 17].

The rate and mode of antimicrobial resistance among these organisms differ from one geographic area to the other. In Lebanon, many studies report a percentage of 3GC-resistance of 40% in *E. coli*, 30% in *Klebsiella spp*, and 15% in *Enterobacter spp* [11, 26–28]. Most of these data come from the compilation of hospital laboratory results with no accurate distinction between community- and hospital-acquired infections. Hospitals in Lebanon have reported a high percentage of 3GC-resistance in nosocomial Enterobacteriaceae infections [28, 29]. Yet, some studies looked at community-acquired isolates and found that the percentage of 3GC-resistance oscillated around 15% [30, 31].

Although the percentage of 3GC-resistant organisms are higher than 10% in the community [30, 31], it would be an overestimation to consider all patients with community-acquired infections as harboring 3GC-resistant Enterobacteriaceae, when considering empiric treatment of cIAI. In fact, patients with community-acquired 3GC-resistant Enterobacteriaceae infections usually have evidence of recent antibiotic exposure [30, 31] or are immunocompromised [30]. Moreover, patients with history of hospitalization, history of surgery or invasive procedures are at significant risk of infection with 3GC-resistant organisms as compared to those who do not those risk factors [30].

On the other hand, among Gram-negative hospital-acquired pathogens implicated in cIAI, the percentage of carbapenem-resistant Enterobacteriaceae (CRE) is rising. In a study of antimicrobial susceptibility data from 13 Lebanese hospital laboratories, the percentage of carbapenem resistance in *E. coli* and *Klebsiella spp.* increased from 0.8 and 2% in 2011–2013 [11] to 3 and 4% in 2015–2016, respectively (unpublished data).

Similarly, Lebanese hospitals have witnessed an emergence of carbapenem-resistant *Acinetobacter baumannii* [32, 33] and *Pseudomonas aeruginosa* during the previous two decades [34, 35]. Some of these organisms have even been reported as extensively resistant to all available antibiotics except colistin with variable resistance to tigecycline [36].

Among the fungal pathogens implicated in cIAI, *Candida* spp. is the most common pathogen. Antifungal susceptibility is not routinely performed in most Lebanese clinical laboratories. In a single medical center in Lebanon, Araj et al. showed an increase in the percentage of *Candida* non-*albicans* among clinically significant *Candida* isolates from 14% in 2005 to 40% in 2014, with emerging resistance to azoles [37]. These concerning data on the emergence of more resistance further support the need for guidance regarding the judicious use of antimicrobial therapy, for optimal outcomes and for prevention of development of yet more resistance.

### Classification of cIAI

Based on anatomical considerations, cIAI are divided in these guidelines into 3 major categories:

1. Peritoneal and intraperitoneal infections that involve the abdomen, bowels, and peritoneum (primary, secondary, and tertiary)
2. Intra-biliary infections, cholecystitis and cholangitis
3. Pancreatitis

Based on microbiological etiology, all types of cIAI share almost the same core organisms; however, there are major differences in the therapeutic rationale among the 3 entities:

- Biliary infections and non-biliary infections have the same causative bacterial organisms; however, the importance of yeast infections is less pronounced in biliary infections compared to non-biliary [20]. In addition, antimicrobial pharmacodynamics parameters differ markedly between the 2 anatomic locations. For example, tigecycline concentration is much higher in the biliary tree compared to the abdominal cavity [38].
- The importance and implication of the bacterial pathogens in the etiology and progression of pancreatitis is different from that in biliary and non-biliary cIAI, and accordingly recommendations for therapy differ.

Another classification is based on two factors considered when selecting antimicrobial agents:

1. Clinical severity measured using severity scores such as the Acute Physiology and Chronic Health Evaluation (APACHE) II score in non-biliary cIAI and cholangitis [1] or grading as in cholecystitis [20].
2. The place where the infection has occurred i.e. in the community or in the hospital/healthcare facility.

### Definitions

- *IAI* are defined as peritoneal inflammation in response to microorganisms, resulting in purulence in the peritoneal cavity. IAIs are classified as uncomplicated or complicated based on the extent of infection [1–3, 19].
- *Uncomplicated IAI* involve a single intra-abdominal organ without anatomical disruption [1–3, 19].
- *cIAI* extend beyond the organ that is the source of the infection, and cause either localized peritonitis, referred to as abdominal abscess, or diffuse peritonitis, depending on the ability of the host to contain the process within a part of the abdominal cavity [1–3, 19]. Hence, cIAI include abdominal cavity infections, biliary infections and pancreatitis.
- *Peritonitis and intraperitoneal bacterial infections* can be classified as primary, secondary or tertiary infections [1–3, 19]:
  - Primary infections refer to spontaneous bacterial invasion of the peritoneal cavity. This mainly occurs in infancy and early childhood, in cirrhotic patients and immunocompromised hosts.
  - Secondary infections describe peritoneal infections secondary to intra-abdominal lesions, such as perforation of the hollow viscus, bowel necrosis, nonbacterial peritonitis, or penetrating infectious processes.
  - Tertiary infections are characterized by persistent or recurrent infections with organisms of low intrinsic virulence or with predisposition for the immunocompromised patient. It usually follows operative attempts to treat secondary peritonitis and is almost exclusively associated with a systemic inflammatory response.
- *Community-acquired cIAI (CA-cIAI)* are defined as infections occurring [1–3, 19]:
  - Prior to hospitalization
  - Within 2 days of hospitalization
- *CA-cIAI with risk of being caused by 3GC-resistant Enterobacteriaceae* should be suspected in patients with [1–3, 19, 30, 31]:
  - Known prior colonization or infection with 3GC-resistant Enterobacteriaceae
  - Exposure to antimicrobials within the previous 90 days
  - History of home infusion therapy (including antibiotics)
  - Home wound care
  - Family member with 3GC-resistant Enterobacteriaceae
  - Immunosuppressive disease and/or therapy
- *Hospital/Health care-associated cIAI (HA-cIAI)* are defined as infections occurring in patients [1–3, 19]:
  - Admitted to the hospital for 48 h or more before the onset of infection, in whom the cIAI had not started before admission
  - Hospitalized for 2 or more days within the preceding 90 days
  - Who are residents of nursing homes or extended care facilities
  - On chronic dialysis

These infections mainly include anastomotic leaks, perforations, and abscesses that develop as a complication of surgery [1, 4].

- *Severity of illness* reflects the risk of mortality in general.
  - Peritoneal/ intraperitoneal infections are stratified to different risk groups (mild to moderate and severe) based on predictable clinical parameters and comorbid conditions as measured by the APACHE II score. Patients with mild to moderate infections are those with an APACHE II score < 15 and those with severe infections have APACHE II score ≥ 15 [4].
  - Similar to peritoneal/intraperitoneal infections, cholangitis is stratified to mild to moderate and severe based on predictable clinical parameters and comorbid conditions as measured by the APACHE II score [4].
  - In cholecystitis, severity of illness is stratified according to the following grading system [20, 39]:
    - *Mild (Grade 1):* Acute cholecystitis that does not meet the criteria for a severe grade: mild gallbladder inflammation and no organ dysfunction.
    - *Moderate (Grade 2):* The presence of one or more of the following parameters:
      - Elevated white-cell count (> 18,000 cells/μL),
      - Palpable tender mass in the upper right abdominal quadrant,
      - Marked local inflammation including biliary peritonitis, pericholecystitic abscess, hepatic abscess, gangrenous cholecystitis, emphysematous cholecystitis,
      - Duration of signs and symptoms > 72 h.
    - *Severe (Grade 3):* The presence of one or more of the following parameters:
      - Cardiovascular dysfunction: hypotension requiring treatment with dopamine at a dose ≥5 mcg/kg/min or any dose of dobutamine,
      - Neurologic dysfunction: decreased level of consciousness,
      - Respiratory dysfunction: ratio of partial pressure of arterial oxygen to the fraction of inspired oxygen < 300,
      - Renal dysfunction: oliguria, serum creatinine level > 2 mg/dL,
      - Hepatic dysfunction: prothrombin time/international normalized ratio (PT/INR) > 1.5,
      - Hematologic dysfunction: platelet count < 100,000/μL.
- *Multi-drug resistant (MDR) organisms (MDRO)* are defined as bacteria which are non-susceptible to at least 1 agent in ≥3 antimicrobial categories [40]. The most commonly described MDRO in these guidelines are 3GC-resistant Enterobacteriaceae that are also resistant to fluoroquinolones and sulfonamides.
- *Extensively-drug resistant (XDR) organisms (XDRO)* are defined as bacteria which are non-susceptible to at least 1 agent in all but 2 or fewer antimicrobials [40]. The most commonly reported XDRO in these guidelines are the carbapenem-resistant *Acinetobacter baumannii*, carbapenem-resistant *Pseudomonas aeruginosa*, and carbapenem-resistant Enterobacteriaceae.

### Diagnosis

Clinical suspicion is based on history and physical examination of the patient, and supported by radiologic, microbiologic, and biochemical evaluation.

#### Radiologic evaluation

Contrast-enhanced computed tomography (CT) scanning is the imaging of choice, except when suspecting biliary tract pathology, and then ultrasound is the modality of choice (grade 2A). When suspicion of cholangitis is high and CT scanning and ultrasound are unrevealing, magnetic resonance cholangiopancreatography (MRCP) is indicated (grade 2A). When immediate laparotomy is indicated such as in septic patients, it should not be delayed while waiting for CT scanning (grade 3A); hence, further diagnostic imaging may be unnecessary in patients with obvious signs of diffuse peritonitis and in whom immediate surgical intervention is to be performed (grade 3B).

#### Microbiologic evaluation

Since source control is the most definitive means of therapeutic intervention, LSIDCM recommends obtaining intra-abdominal cultures whenever possible because of the reported prevalence of MDR Enterobacteriaceae in data published from Lebanon (grade 3A). Additionally, blood cultures are recommended for all patients upon presentation, prior to initiation of antimicrobial therapy.

#### Biochemical evaluation

For inpatients suspected of having cIAI, the following tests are required: complete blood count and differential, full biochemical profile (including renal and hepatic panels), and C-reactive protein (CRP) (grade 3A). The role of procalcitonin (PCT) in cIAI is not clear yet, although emerging data support its application in clinical scenarios of severe infection to guide duration and assess response to antibiotic therapy [3, 41]. The LSIDCM suggests judicious utilization of biomarkers (PCT, CRP, lactate level) as aids in the management of cIAI including prediction of severity and response to antimicrobial therapy (grade 3A).

#### Others

For selected patients with unreliable physical examination findings, such as those with obtunded mental status, spinal cord injury, and immune compromised status, IAI should be considered if these patients present with evidence of infection from an undetermined source (grade 3B).

### Treatment

#### Principles of therapy

The three main pillars of therapy in cIAI are source control, antimicrobial therapy and hemodynamic restoration (grade 1).

Source control through surgery is recommended whenever possible and as soon as possible (grade 2A), such as in patients with diffuse peritonitis (grade 2A). Laparoscopic versus open procedures are the surgeon’s choice. The details of these surgical interventions are beyond the scope of these guidelines.

Antimicrobial therapy should be initiated within the first hour of presentation when patients are septic (2A), and within the first 8 h when they are clinically stable as work-up is being completed and a diagnosis is being formulated (grade 2B).

In patients with septic shock, the LSIDCM recommends following the Surviving Sepsis Campaign Guidelines [42], with a goal of restoring hemodynamic stability, such as giving 30 mL of crystalloid per kg body weight within the first 3 h of presentation (grade 3A). Individualized parameters such as filling pressure, oxygen saturation, and other functional hemodynamic measures should be utilized to achieve customized resuscitation in the setting of sepsis from cIAI (grade 3A).

The role of corticosteroids is limited. Stress-dose steroid therapy is considered only in septic shock when blood pressure is poorly responsive to fluid and vasopressor therapy (grade 3A). The preferred agent is intravenous hydrocortisone at a dose of 200 mg per day.

#### Antibiotic therapy recommendations for peritoneal and intraperitoneal infections

Although the causative organisms in cIAI are the same in community-acquired and in nosocomial settings, the antibiotic susceptibility patterns of these organisms differ vastly.

For community-acquired infections, we divided patients with or without risk factors for the acquisition of 3GC-resistant Enterobacteriaceae.

In hospitals and other healthcare institutions, based on the local epidemiology, all patients are at risk of nosocomial acquisition of 3GC-resistant Enterobacteriaceae ([11, 26] unpublished data). In addition, XDRO like XDR *Pseudomonas aeruginosa*, XDR *Acinetobacter baumannii*, and carbapenem-resistant Enterobacteriaceae are more likely to be causatives in cIAI of hospital onset. Several XDRO have been reported endemic in different healthcare institutions in Lebanon [32–36, 43, 44].

To avoid using last-line antibiotics empirically in all patients, the LSIDCM panel recommends initiating broad-spectrum antibiotics that cover 3GC-resistant Enterobacteriaceae in community-acquired infections or cover XDRO in hospital-acquired infections in critically ill patients, then de-escalate antibiotic therapy based on culture results. On the other hand, in clinically stable patients, we advise initiation of a relatively narrow-spectrum antibiotic regimen as empiric therapy, and then change according to culture results.

Accordingly, our recommendations are stratified based on the clinical condition of the patient and the site of onset of the IAI. It is worth mentioning that no international guidelines described treatment recommendations for HA-infections that are compatible with the antibiotic resistance trends in Lebanese hospitals. Thus, our recommendations are based on expert opinion.

For HA-infections, hospitals were classified as follows:

- *Group A Hospitals*: have more than 20% 3GC-resistance in nosocomial Enterobacteriaceae and less than 20% resistance to ceftazidime and carbapenems in nosocomial *Pseudomonas aeruginosa*,
- *Group B Hospitals*: have more than 20% 3GC-resistance in nosocomial Enterobacteriaceae and more than 20% resistance to carbapenems in *Acinetobacter baumannii*,
- *Group C Hospitals*: have more than 20% 3G-resistance in nosocomial Enterobacteriaceae and more than 20% resistance to ceftazidime and carbapenems in nosocomial *Pseudomonas aeruginosa*,
- *Group D Hospitals*: have more than 20% resistance to 3GC and carbapenems in nosocomial Enterobacteriaceae.

Understandably, some hospitals have a combination of XDRO in their ecology, or other types of organisms. Therefore, these guidelines cannot cover all the possibilities, but do provide a general reference to the approach of managing these infections in different nosocomial settings.

Recommendations for the empiric antimicrobial treatment of community-acquired peritoneal and intraperitoneal infections are summarized in Table 2. As for hospital-acquired peritoneal and intraperitoneal infections, recommendations per hospital type (A, B, C or D) are presented in Tables 3 and 4.

**Table 2:** Empiric antimicrobial therapy for community-acquired complicated intra-abdominal infections

Type of infection	Classification	Sub-classification	Duration of antimicrobial therapy	Recommendation
Peritoneal/ Intra-peritoneal	*Mild to moderate with hemodynamic stability (no spillage of intraluminal material in the peritoneum)*	Acute stomach or duodenal/ proximal jejunal perforation in the absence of gastric acid-reducing therapy or malignancy, and when the patient is operated within 24 h	24 h (grade 3A)	*No risk of 3GCRE* -[AMC + AMG (grade 2B)] Or -[3GC (CRO or CTX or ZOX) + MTZ ± AMG (grade 2B)] -No additional antibiotic coverage against *Enterococci* (grade 1) -No additional antifungal coverage (grade 2B) *Risk of 3GCRE* -ETP (grade 1) or TGC (grade 2B) -No additional antibiotic coverage against *Enterococci* (grade 1) -No additional antifungal coverage (grade 2B)
		Bowel injuries attributed to penetrating, blunt, or iatrogenic trauma repaired within 12 h without any intraoperative contamination of the operative field by enteric contents	24 h (grade 1)	
		Acute appendicitis without evidence of perforation, abscess, local peritonitis, or spillage of intraluminal material in the peritoneum	24 h (grade 1)	
	*Mild to moderate with hemodynamic stability (with intra-abdominal contamination with intraluminal material)*	Acute stomach or duodenal/ proximal jejunal perforation in case of delayed operation > 24 h, the presence of gastric malignancy or the presence of therapy reducing gastric acidity and the infection is ongoing or persistent	4–7 d^1^ (grade 3B)	*No risk of 3GCRE* -[AMC + AMG (grade 2B)] Or -[3GC (CRO or CTX or ZOX) + MTZ ± AMG (grade 2B)] -No additional antibiotic coverage against *Enterococci* (grade 1) -No additional antifungal coverage (grade 2B) *Risk of 3GCRE* -ETP (grade 1) or TGC (grade 2B) -No additional antibiotic coverage against *Enterococci* (grade 1) -No additional antifungal coverage (grade 2B)
		Bowel injuries attributed to penetrating, blunt, or iatrogenic trauma repaired within 12 h (with intra-abdominal contamination with intraluminal material)	4–7 d^1^ (grade 3B)	
		Acute appendicitis (with intra-abdominal contamination with intraluminal material)	4–7 d^1^ (grade 3B)	
	*Severe (Appendicitis, colonic non-diverticular perforation, diverticulitis, gastro-duodenal perforations, small bowel perforation, pelvic inflammatory disease, post-traumatic perforation)*	No secondary bacteremia; Adequate source control	4 d^1^ (grade 2A)	- CAR (IPM or MEM) (grade 1) - Use glycopeptides for *Enterococci* treatment in immunocompromised patients or those with recurrent infection (grade 2B) - Antifungal therapy: • FLC as targeted therapy in high risk^2^, non-septic, immunocompetent patients (grade 2B) • Echinocandins (AFG, CAS or MFG) in septic or immunocompromised patients (grade 3B)
		Secondary bacteremia; Adequate source control with successful treatment of bacteremia	7 d^1^ (grade 2B)	
		No adequate source control	> 7–14 d^1^ (grade 3B)	
Cholecystitis	*Grade 1*	–	24 h (grade 1)	AMC (grade 2B) or CXM (grade 2B) or 3GC (CRO or CTX or ZOX) (grade 2A)
	*Grade 2*	–	4–7 d (grade 3B) (adequate source control)	*No risk of 3GCRE* 3GC (CRO or CTX or ZOX) + MTZ (grade 2A) *Risk of 3GCRE* ETP (1) or TGC (2B) or TZP (3B)
	*Grade 3*	–	≥ 5 d ^3^ (grade 3B)	CAR (IPM or MEM) (1)
Cholangitis	*Mild to moderate*	–	4–7 d ^4^ (grade 3B) (adequate source control)	*No risk of 3GCRE* 3GC (CRO or CTX or ZOX) + MTZ (grade 2A) *Risk of 3GCRE* ETP (grade 1) or TGC (grade 2B) or TZP (grade 3B)
	*Severe* (*including perforation, emphysema, and necrosis of gall bladder,* etc.*)*	–	≥ 5 d ^3,4^ (grade 3B)	CAR (IPM or MEM) + glycopeptide (grade 1)

*KEY: AFG* anidulafungin, *AMC* amoxicillin/clavulanic acid, *AMG* aminoglycoside, *CAS* caspofungin, *CRO* ceftriaxone, *CTX* cefotaxime, *CXM* cefuroxime, *d* days, *ETP* ertapenem, *FLC* fluconazole, *h* hours, *IPM* imipenem, *MEM* meropenem, *MFG* micafungin, *MTZ* metronidazole, *TGC* tigecycline, *TZP* piperacillin/tazobactam, *XDRO* extensively-drug resistant organism, *ZOX* ceftizoxime, *3GC* third generation cephalosporin, *3GCRE* third generation cephalosporin-resistant Enterobacteriaceae

N.B

^1^The decision to continue, revise, or stop antimicrobial therapy should be made on the basis of clinician judgment and laboratory information (grade 3A). Criteria to evaluate clinical efficacy and duration of antimicrobial therapy are presence of comorbidities, quality of the surgical procedure, time to apyrexia, normalization of leukocyte count, normalization of bowel movements. Severity and correction of organ failure are criteria for evaluating treatment efficacy severe infections only

^2^High-risk patients include those with advanced age, high disease severity and high risk of death

^3^Duration extended depending on: severity of organ failure, concomitant presence of bacteremia, rate of resolution of fever and leukocytosis, and correction of organ failure

^4^If residual stones or obstruction of the bile tract are present, treatment should be continued until these anatomical problems are resolved (grade 3B)

**Table 3:** Empiric antimicrobial therapy for hospital-acquired complicated intra-abdominal infections (Hospitals groups A and B)

Type of infection	Classification	Sub-classification	Hospitals Group A (> 20% 3GC resistance in nosocomial Enterobacteriaceae and < 20% resistance to CAZ and CAR in nosocomial *P. aeruginosa*		Hospitals Group B (> 20% 3GC resistance in nosocomial Enterobacteriaceae and > 20% resistance to CAR in *A. baumannii*)	
			Duration of antimicrobial therapy	Recommendation	Duration of antimicrobial therapy	Recommendation
Peritoneal/ Intra-peritoneal	*Mild to moderate with hemodynamic stability (no spillage of intraluminal material in the peritoneum)*	Acute stomach or duodenal/ proximal jejunal perforation in the absence of gastric acid-reducing therapy or malignancy, and when the patient is operated within 24 h	5 d (grade 2B)	1- CAR sparing regimen: [(TZP or CAZ or FEP) + TGC] (grade 3B) + Echinocandin (AFG, CAS or MFG) (grade 2A) Or 2- [CAR (IPM or MEM) (grade 3C) + anti-MRSA antibiotics^2^ (grade 2A)] + Echinocandin (AFG, CAS or MFG) (grade 2A)	7 d (grade 2B)	If the patient was not on a CAR-containing regimen: [CAR (IPM or MEM) (grade 3C) + anti-MRSA antibiotics^1^ (grade 2A)] + Echinocandin (AFG, CAS or MFG) (grade 2A) If the patient was on a CAR containing regimen: [(C/T or TZP or CAZ or FEP) + TGC] (grade 3C) + Echinocandin (AFG, CAS or MFG) (grade 2A) *In case of positive XDRO screen, modify antibacterial regimen as per culture results.
		Bowel injuries attributed to penetrating, blunt, or iatrogenic trauma repaired within 12 h without any intraoperative contamination of the operative field by enteric contents	5 d grade 2B)		7 d (grade 2B)	
		Acute appendicitis without evidence of perforation, abscess, local peritonitis, or spillage of intraluminal material in the peritoneum	5 d (grade 2B)		7 d (grade 2B)	
	*Mild to moderate with hemodynamic stability (with intra-abdominal contamination with intraluminal material)*	Acute stomach or duodenal/ proximal jejunal perforation in case of delayed operation > 24 h, the presence of gastric malignancy or the presence of therapy reducing gastric acidity and the infection is ongoing or persistent	7–10 d^2^ (grade 2B)	1- CAR sparing regimen: [(TZP or CAZ or FEP) + TGC] (grade 3B) + Echinocandin (AFG, CAS or MFG) (grade 2A) Or 2- [CAR (IPM or MEM) (grade 3C) + anti-MRSA antibiotics^1^ (grade 2A)] + Echinocandin (AFG, CAS or MFG) (grade 2A)	7–14 d^2^ (grade 2B)	If the patient was not on a CAR-containing regimen: [CAR (IPM or MEM) (grade 3C) + anti-MRSA antibiotics^1^ (grade 2A)] + Echinocandin (AFG, CAS or MFG) (grade 2A) If the patient was on a CAR containing regimen: [(TZP or CAZ or FEP) + TGC] (grade 3C) + Echinocandin (AFG, CAS or MFG) (grade 2A) *In case of positive XDRO screen, modify antibacterial regimen as per culture results.
		Bowel injuries attributed to penetrating, blunt, or iatrogenic trauma repaired within 12 h (with intra-abdominal contamination with intraluminal material)	7–10 d^2^ (grade 2B)		7–14 d^2^ (grade 2B)	
		Acute appendicitis (with intra-abdominal contamination with intraluminal material)	7–10 d^2^ (grade 2B)		7–14 d^2^ (grade 2B)	
	*Severe (Appendicitis, colonic non-diverticular perforation, diverticulitis, gastro-duodenal perforations, small bowel perforation, pelvic inflammatory disease, post-traumatic perforation)*	No secondary bacteremia; Adequate source control	7–14 d^2^ (grade 2B)	1- [CAR (IPM or MEM) (grade 1) + Glycopeptide or LZD^3^ (grade 2B)] + Echinocandin (AFG, CAS or MFG) (grade 2A) Or 2-CAR sparing regimen: [(C/T + MTZ) or (CZA + MTZ) (grade 2B) + Glycopeptide or LZD^3^ (grade 2B)] + Echinocandin (AFG, CAS or MFG) (grade 2A)	7–14 d^2^ (grade 2B)	1-[CAR (IPM or MEM) + CST + Glycopeptide or LZD^3^ (grade 3C)] + Echinocandin (AFG, CAS or MFG) (grade 2A) Or 2- CAR sparingregimen: [(TZP or CAZ or FEP) + CST + TGC + Glycopeptide or LZD^3^ (grade 3C)] + Echinocandin (AFG, CAS or MFG) (grade 2A) *In case of negative XDRO screen or intra-operative cultures for *Acinetobacter* spp., discontinue colistin.
		Secondary bacteremia; Adequate source control with successful treatment of bacteremia	10–14 d^2^ (grade 2B)		10–14 d^2^ (grade 2B)	
		No adequate source control	> 10–14 d^2^ (grade 2B)		≥ 14 d^2^ (grade 2B)	
Cholecystitis	*Grade 1*	–	4 d (grade 2B)	1- CAR sparing regimen: [(TZP or CAZ or FEP) + TGC] (grade 3B) Or 2- [CAR (IPM or MEM) (grade 3C) + anti-MRSA antibiotics^1^ (grade 2A)]	5 d (grade 2B)	If the patient was not on a CAR-containing regimen: [CAR (IPM or MEM) (grade 3C) + anti-MRSA antibiotics^1^ (grade 2A)] If the patient was on a CAR containing regimen: [(TZP or CAZ or FEP) + TGC] (grade 3C) *In case of positive XDRO screen, modify antibacterial regimen as per culture results.
	*Grade 2*	–	7–10 d (grade 2B) (adequate source control)		7–10 d (grade 2B) (adequate source control)	
	*Grade 3*	–	≥ 10 d^4^ (grade 2B)		≥ 10–14 d^4^ (grade 2B)	
Cholangitis^5,6^	*Mild to moderate*	–	> 7 d^4^ (grade 3B)	1- CAR sparing regimen: [(TZP or CAZ or FEP) + TGC] (grade 3B) Or 2- [CAR (IPM or MEM) (grade 3C) + anti-MRSA antibiotics^1^ (grade 2A)]	7–10 d (grade 2B)	If the patient was not on a CAR-containing regimen: [CAR (IPM or MEM) (grade 3C) + anti-MRSA antibiotics^1^ (grade 2A)] If the patient was on a CAR containing regimen: [(TZP or CAZ or FEP) + TGC] (grade 3C) *In case of positive XDRO screen, modify antibacterial regimen as per culture results.
	*Severe* (*including perforation, emphysema, and necrosis of gall bladder,* etc.*)*	–	≥ 10 d^4^ (grade 2B)		≥ 10–14 d^4^ (grade 2B)	

*KEY: AFG* anidulafungin, *AMK* amikacin, *CAS* caspofungin, *CAZ* ceftazidime, *CST* colistin, *CZA* ceftazidime/avibactam, *C/T* ceftolozane/tazobactam, *d* days, *DD* double dose, *ETP* ertapenem, *FEP* cefepime, *h* hours, *IPM* imipenem, *LZD* linezolid, *MEM* meropenem, *MFG* micafungin, *MIC* minimal inhibitory concentration, *MRSA* Methicillin-resistant *Staphylococcus aureus*, *MTZ* metronidazole, *TGC* tigecycline, *TZP* piperacillin/tazobactam, *XDRO* Extensively-drug resistant organism

N.B.*Screening for XDRO carriage is recommended in Hospitals B, C and D (grade 2B)

^1^Risk factors for MRSA acquisition include: history of previous colonization, history of close proximity to cases harboring/infected with MRSA, prior treatment failure due to an MRSA-related infection, and extensive exposure to antibiotics

^2^The decision to continue, revise, or stop antimicrobial therapy should be made on the basis of clinician judgment and laboratory information (grade 3A). Criteria to evaluate clinical efficacy and duration of antimicrobial therapy are presence of comorbidities, quality of the surgical procedure, time to apyrexia, normalization of leukocyte count, normalization of bowel movements. Severity and correction of organ failure are criteria for evaluating treatment efficacy severe infections only

^3^Linezolid should be used in patients at risk of vancomycin-resistant Enterococci (VRE)-related infection only. Risk factors for VRE include: previous antibiotic therapy, prolonged hospitalization, hospitalization in an intensive care unit, severe illness or underlying pathology, invasive procedures, gastrointestinal surgery, organ transplantation, and close proximity to other VRE-positive patients

^4^The duration of antimicrobial therapy is extended depending on concomitant presence of bacteremia and rate of resolution of fever and leukocytosis. Severity and correction of organ failure are criteria for treatment efficacy in severe cases only

^5^In case of post-operative cholangitis or cholangitis complicated by septic shock, add an echinocandin (AFG, CAS or MFG) to the antibiotic regimen (grade 2A)

^6^If residual stones or obstruction of the bile tract are present, treatment should be continued until these anatomical problems are resolved

**Table 4:** Empiric antimicrobial therapy for hospital-acquired complicated intra-abdominal infections (Hospitals groups C and D)

Type of infection	Classification	Sub-classification	Hospitals Group C (> 20% 3G resistance in nosocomial Enterobacteriaceae and > 20% resistance to CAZ and CAR in nosocomial *P. aeruginosa)*		Hospitals Group D (> 20% resistance to 3GC and CAR in nosocomial Enterobacteriaceae	
			Duration of antimicrobial therapy	Recommendation	Duration of antimicrobial therapy	Recommendation
Peritoneal/ Intra-peritoneal	*Mild to moderate with hemodynamic stability* *(No spillage of intraluminal material in the peritoneum)*	Acute stomach or duodenal/ proximal jejunal perforation in the absence of gastric acid-reducing therapy or malignancy, and when the patient is operated within 24 h	7 d (grade 2B)	If the patient was not on a CAR-containing regimen: [CAR (IPM or MEM) (grade 3C) + anti-MRSA antibiotics^1^ (grade 2A)] + Echinocandin (AFG, CAS or MFG) (grade 2A) If the patient was on a CAR containing regimen: [(TZP or CAZ or FEP) + TGC] (grade 3C) + Echinocandin (AFG, CAS or MFG) (grade 2A) Or [C/T (grade 3C) + anti-MRSA antibiotics^1^ (grade 2A)] + Echinocandin (AFG, CAS or MFG) (grade 2A) *In case of positive XDRO screen, modify antibacterial regimen as per culture results.	7 d (grade 2B)	If the patient was not on a CAR-containing regimen: [CAR (IPM or MEM) (grade 3C) + anti-MRSA antibiotics^1^ (grade 2A)] + Echinocandin (AFG, CAS or MFG) (grade 2A) If the patient was on a CAR containing regimen: [(TZP or CAZ or FEP) + TGC] (grade 3C) + Echinocandin (AFG, CAS or MFG) (grade 2A) Or [C/T (grade 3C) + anti-MRSA antibiotics^1^ (grade 2A)] + Echinocandin (AFG, CAS or MFG) (grade 2A) *In case of positive XDRO screen, modify antibacterial regimen as per culture results..
		Bowel injuries attributed to penetrating, blunt, or iatrogenic trauma repaired within 12 h without any intraoperative contamination of the operative field by enteric contents	7 d (grade 2B)		7 d (grade 2B)	
		Acute appendicitis without evidence of perforation, abscess, local peritonitis, or spillage of intraluminal material in the peritoneum	7 d (grade 2B)		7 d (grade 2B)	
	*Mild to moderate with hemodynamic stability (with intra-abdominal contamination with intraluminal material)*	Acute stomach or duodenal/ proximal jejunal perforation in case of delayed operation > 24 h, the presence of gastric malignancy or the presence of therapy reducing gastric acidity and the infection is ongoing or persistent	7–14 d^2^ (grade 2B)	If the patient was not on a CAR-containing regimen: [CAR (IPM or MEM) (grade 3C) + anti-MRSA antibiotics^1^ (grade 2A)] + Echinocandin (AFG, CAS or MFG) (grade 2A) If the patient was on a CAR containing regimen: [(TZP or CAZ or FEP) + TGC] (grade 3C) + Echinocandin (AFG, CAS or MFG) (grade 2A) Or [C/T (grade 3C) + anti-MRSA antibiotics^1^ (grade 2A)] + Echinocandin (AFG, CAS or MFG) (grade 2A) *In case of positive XDRO screen, modify antibacterial regimen as per culture results.	7–14 d^2^ (grade 2B)	If the patient was not on a CAR-containing regimen: [CAR (IPM or MEM) (grade 3C) + anti-MRSA antibiotics^1^ (grade 2A)] + Echinocandin (AFG, CAS or MFG) (grade 2A) If the patient was on a CAR containing regimen: [(TZP or CAZ or FEP) + TGC] (grade 3C) + Echinocandin (AFG, CAS or MFG) (grade 2A) Or [C/T (grade 3C) + anti-MRSA antibiotics^1^ (grade 2A)] + Echinocandin (AFG, CAS or MFG) (grade 2A) *In case of positive XDRO screen, modify antibacterial regimen as per culture results.
		Bowel injuries attributed to penetrating, blunt, or iatrogenic trauma repaired within 12 h (with intra-abdominal contamination with intraluminal material)	7–14 d^2^ (grade 2B)		7–14 d^2^ (grade 2B)	
		Acute appendicitis (with intra-abdominal contamination with intraluminal material)	7–14 d^2^ (grade 2B)		7–14 d^2^ (grade 2B)	
	*Severe (Appendicitis, colonic non-diverticular perforation, diverticulitis, gastro-duodenal perforations, small bowel perforation, pelvic inflammatory disease, post-traumatic perforation)*	No secondary bacteremia; Adequate source control	7–14 d^2^ (grade 2B)	CAR sparing regimen: [C/T + MTZ + AMK ± CST (grade 2B) + Glycopeptide or LZD^3^ (grade 3C)] + Echinocandin (AFG, CAS or MFG) (grade 2A) If C/T is not available: 1- CAR sparing regimen: [(TZP or CAZ or FEP) + CST + TGC + Glycopeptide or LZD^3^ (grade 3C)] + Echinocandin (AFG, CAS or MFG) (grade 2A) Or 2- [CAR (IPM or MEM) + CST + Glycopeptide or LZD^3^ (grade 3C)] + Echinocandin (AFG, CAS or MFG) (grade 2A) *In case of negative XDRO screen or intra-operative cultures for *Pseudomonas* spp., discontinue colistin.	7–14 d^2^ (grade 2B)	If MEM MIC ≤ 16 μg/mL: 1-[MEM (DD and prolonged infusion over 4 h) + TGC ± AMK (grade 3C) + Glycopeptide or LZD^3^ (grade 2A)] + Echinocandin (AFG, CAS or MFG) (grade 2A) Or 2- CAR sparing regimen: [CZA + TGC ± AMK (grade 2B) + Glycopeptide or LZD^3^ (grade 2A)] + Echinocandin (AFG, CAS or MFG) (grade 2A) If MEM MIC > 16 μg/mL: 1-[CZA + TGC ± AMK (grade 2B) + Glycopeptide or LZD^3^ (grade 2A)] + Echinocandin (AFG, CAS or MFG) (grade 2A) Or 2- [dual CAR regimen (MEM + ETP) + CST + TGC ± AMK (grade 3C) + Glycopeptide or LZD^3^ (grade 2A)] + Echinocandin (AFG, CAS or MFG) (grade 2A) *In case of negative XDRO screen or intra-operative cultures for CRE, discontinue colistin.
		Secondary bacteremia; Adequate source control with successful treatment of bacteremia	10–14 d^2^ (grade 2B)		10–14 d^2^ (grade 2B)	
		No adequate source control	≥ 14 d^2^ (grade 2B)		≥ 14 d^2^ (grade 2B)	
Cholecystitis	*Grade 1*	–	5 d (grade 2B)	1- CAR sparing regimen: [(TZP or CAZ or FEP) + TGC] (grade 3B) Or 2- [CAR (IPM or MEM) (grade 3C) + anti-MRSA antibiotics^1^ (grade 2A)]	5 d (grade 2B)	If the patient was not on a CAR-containing regimen: [CAR (IPM or MEM) (grade 3C) + anti-MRSA antibiotics^1^ (grade 2A)] If the patient was on a CAR containing regimen: [(TZP or CAZ or FEP) + TGC] (grade 3C) *In case of positive XDRO screen, modify antibacterial regimen as per culture results.
	*Grade 2*	–	7–10 d (grade 2B) (adequate source control)		7–10 d (grade 2B) (adequate source control)	
	*Grade 3*	–	≥ 10–14 d^4^ (grade 2B)		≥ 10–14 d^4^ (grade 2B)	
Cholangitis^5,6^	*Mild to moderate*	–	7–10 d (grade 2B)	1- CAR sparing regimen: [(TZP or CAZ or FEP) + TGC] (grade 3B) Or 2- [CAR (IPM or MEM) (grade 3C) + anti-MRSA antibiotics^1^ (grade 2A)]	7–10 d (grade 2B)	If the patient was not on a CAR-containing regimen: [CAR (IPM or MEM) (grade 3C) + anti-MRSA antibiotics^1^ (grade 2A)] If the patient was on a CAR containing regimen: [(TZP or CAZ or FEP) + TGC] (grade 3C) *In case of positive XDRO screen, modify antibacterial regimen as per culture results.
	*Severe* (*including perforation, emphysema, and necrosis of gall bladder,* etc.*)*	–	≥ 10–14 d^4^ (grade 2B)		≥ 10–14 d^4^ (grade 2B)	

*KEY: AFG* anidulafungin, *AMK* amikacin, *CAS* caspofungin, *CAZ* ceftazidime, *CST* colistin, *CZA* ceftazidime/avibactam, *C/T* ceftolozane/tazobactam, *d* days, *DD* double dose, *ETP* ertapenem, *FEP* cefepime, *h* hours, *IPM* imipenem, *LZD* linezolid, *MEM* meropenem, *MFG* micafungin, *MIC* minimal inhibitory concentration, *MRSA* Methicillin-resistant *Staphylococcus aureus*, *MTZ* metronidazole, *TGC* tigecycline, *TZP* piperacillin/tazobactam, *XDRO* Extensively-drug resistant organism

N.B.*Screening for XDRO carriage is recommended in Hospitals B, C and D (grade 2B)

^1^Risk factors for MRSA acquisition include: history of previous colonization, history of close proximity to cases harboring/infected with MRSA, prior treatment failure due to an MRSA-related infection, and extensive exposure to antibiotics

^2^The decision to continue, revise, or stop antimicrobial therapy should be made on the basis of clinician judgment and laboratory information (grade 3A). Criteria to evaluate clinical efficacy and duration of antimicrobial therapy are presence of comorbidities, quality of the surgical procedure, time to apyrexia, normalization of leukocyte count, normalization of bowel movements. Severity and correction of organ failure are criteria for evaluating treatment efficacy severe infections only

^3^Linezolid should be used in patients at risk of vancomycin-resistant Enterococci (VRE)-related infection only. Risk factors for VRE include: previous antibiotic therapy, prolonged hospitalization, hospitalization in an intensive care unit, severe illness or underlying pathology, invasive procedures, gastrointestinal surgery, organ transplantation, and close proximity to other VRE-positive patients

^4^The duration of antimicrobial therapy is extended depending on concomitant presence of bacteremia, rate of resolution of fever and leukocytosis, in addition to severity and correction of organ failure

^5^In case of post-operative cholangitis or cholangitis complicated by septic shock, add an echinocandin (AFG, CAS or MFG) to the antibiotic regimen (grade 2A)

^6^If residual stones or obstruction of the bile tract are present, treatment should be continued until these anatomical problems are resolved

#### Antimicrobial therapy recommendations for biliary tract infections

Patients are classified as having community- or hospital-acquired cholecystitis or cholangitis. Cholecystitis is further stratified according to a specific grading of severity (grades 1, 2 and 3). Cholangitis severity is divided into mild to moderate and severe infection based on the APACHE II score. Empiric treatment recommendation for community-acquired cholecystitis and cholangitis are summarized in Table 2 and those of hospital-acquired cholecystitis and cholangitis in Tables 3 and 4.

For all conditions, refer to Table 6 for antimicrobial dosing.

### Acute pancreatitis

#### Diagnosis

The diagnosis of acute pancreatitis (AP) is based on the fulfillment of 2 out of 3 of the following criteria: clinical (upper abdominal pain), laboratory (serum amylase or lipase.

>3x upper limit of normal) and/or imaging criteria (CT, magnetic resonance imaging, ultrasonography) (grade 2A).

#### Classification and complications

*The level of AP severity based on Atlanta classification is classified as follows:*

- *Mild AP*: no organ failure, local or systemic complications,
- *Moderately severe AP*: organ failure that resolves within 48 h and/or local or systemic complications without persistent organ failure,
- *Severe AP*: persistent organ failure > 48 h,
- *Interstitial edematous AP*: acute inflammation of the pancreatic parenchyma and peri-pancreatic tissues, but without recognizable tissue necrosis,
- *Necrotizing AP*: inflammation associated with pancreatic parenchymal necrosis and/or peri-pancreatic necrosis. Infected pancreatic necrosis should be considered when the following conditions are present: the necrosis is extensive involving 30% or more of the pancreas, the patient fails to improve, or deteriorates, after 7 to 10 days of appropriate in-hospital care for acute pancreatitis, along with the development of gas in the area of pancreatic necrosis, all this being associated with rising inflammatory markers or persistent fever.

*Complications of AP are:*

- Organ failure and other systemic complications
  - Respiratory: PaO_2_/FiO_2_ ≤ 300
  - Cardiovascular: systolic blood pressure < 90 mmHg (off inotropic support), not fluid responsive, or pH < 7.3
  - Renal: serum creatinine ≥1.9 mg/dL (170 μmol/L)
- Local complications
  - Acute peri-pancreatic fluid collections
  - Pancreatic pseudocysts
  - Acute necrotic collections
  - Walled-off pancreatic necrosis

#### Management

The mainstay therapy in AP is fluid resuscitation using crystalloids (grade 2A). Intravenous fluid therapy with 5–10 mL/kg/h should be used initially until resuscitation goals are reached. The aim of fluid resuscitation is to reach a heart rate of < 120/min, mean arterial pressure between 65 and 85 mmHg (8.7–11.3 kPa), and urinary output > 0.5-1 mL/kg/h (grade 2B).

In case of biliary pancreatitis, early endoscopic retrograde cholangiopancreatography/ endoscopic sphincterotomy (ERCP/ES) should be performed in gallstone-induced AP when complications of cholangitis or prolonged passage disorder of the biliary tract are suspected (grade 1). To prevent the recurrence of gallstone-induced AP, cholecystectomy is recommended for cases where such surgery is possible (grade 2A). Cholecystectomy should be performed as soon as gallstone-induced AP has resolved (grade 2A).

Severity assessment is recommended immediately after diagnosis and repeated over time (especially within 48 h of the diagnosis) (grade 3A), using a specific scoring system (grade 2A). Even when the case is mild in its early stages, severity assessment should be carried out repeatedly over time, and when higher severity criteria are met, transfer to an intensive care unit should be considered (grade 3A).

#### Antimicrobial therapy considerations (Tables 5 and 6)

- The decision to give antimicrobials depends upon the severity and complications of AP.
- The choice of antimicrobials is based upon tissue penetration of the antimicrobial inside the pancreas and susceptibility of the infecting organism to the chosen antimicrobial.
- Any concomitant extrapancreatic infection, such as, cholangitis, cholecystitis, pneumonia, urinary tract infection, should be promptly treated with antimicrobials (grade 3B).
- The prophylactic administration of antibiotics is not necessary in mild AP, since the incidence and mortality rates of infectious complications from mild AP are low (grade 2A). (Table 5)
- The prophylactic administration of antibiotics in severe AP is recommended in the early disease stages (within 72 h of onset) (grade 2B). (Table 5)
- Antimicrobial therapy is indicated in infected pancreatic necrosis (grade 3B). In this case, initial CT-guided fine needle aspiration (FNA) for Gram stain and culture to guide use of appropriate antibiotics is desirable; alternatively, empiric use of antibiotics should be provided if there is no access to CT FNA (grade 3B).
- Carbapenems and fluoroquinolones have the best penetration into the pancreatic tissue among antibiotics active against Enterobacteriaceae [45, 46]. Piperacillin/tazobactam has an acceptable pancreatic tissue penetration [47], while both cephalosporins and aminoglycosides have poor pancreatic tissue penetration [46].
- Based on antimicrobial resistance issues discussed earlier in these guidelines, fluoroquinolones are not recommended as empiric treatment in severe pancreatitis and infected pancreatic necrosis (grade 3B). Instead, carbapenems (imipenem or meropenem) are recommended for empiric therapy in the above stated indications (grade 3B). In cases of proven susceptibility of the recovered organisms to fluroquinolones or piperacillin/tazobactam, then these agents are recommended as targeted, carbapenem-sparing therapy (grade 3B). (Table 5)
- Routine administration of antifungals is not recommended in AP (grade 3B). This is considered only in case of no response to antibiotics, presence of confirmed infection due to *Candida spp.*, or risk factors for *Candida* spp. infection.
- Probiotic administration is not recommended for the prevention of infectious complications in AP (grade 2A).
- Therapeutic intervention for infected pancreatic necrosis should be performed after 4 weeks of onset, if possible, when the necrosis has been sufficiently walled off (grade 3B). Details of the invasive intervention in pancreatic necrosis are beyond the scope of this manuscript.

**Table 5:** Antimicrobial therapy in acute pancreatitis

Classification	Duration of antimicrobial therapy	Recommendation
Mild	No antibiotics (grade 2A)	No antibiotics (grade 2A)
*Severe*	Up to 14 d (grade 3B)	-Empiric therapy: CAR (IPM or MEM) (grade 3B) -CAR sparing regimen: TZP or FQ (if proven antibiotic susceptibility of the recovered organisms) (grade 3B)
*Infected pancreatic necrosis*		

*KEY: CAR* carbapenems, *d* days, *FQ* fluoroquinolone, *IPM* imipenem, *MEM* meropenem, *TZP* piperacillin/tazobactam

**Table 6:** Dosing of antimicrobials used in the management of intra-abdominal infections in adults with normal renal function

Antimicrobials	Dose
β-lactam/β-lactamase inhibitor combination	
AMC	2.2 g IV every 6 h; 2 h infusion time^a^
TZP	4.5 g IV every 6 h; 3 h infusion time
C/T	1.5 g IV every 8 h
CZA	2.5 g IV every 8 h
Carbapenems	
ETP	1 g IV every 24 h, consider 2 h infusion time
IPM	1 g IV every 8 h
MEM	1 g IV every 8 h; consider 2 g IV loading dose; 4 h hour infusion time
Cephalosporins	
CXM	1.5 g IV every 8 h
CRO	2 g IV every 12–24 h
CTX	1–2 g IV every 6–8 h
ZOX	1–2 g IV every 8–12 h
FEP	2 g IV every 8 h
CAZ	2 g IV every 8 h
Glycylcyclines	
TGC	100 mg IV loading dose, then 50 mg IV every 12 h
Polymyxin	
Colistimethate sodium	9 million IU IV loading dose, then 4.5 million IU IV q12 h
Aminoglycosides	
GEN	5–7 mg/kg IV every 24 h
AMK	15–20 mg/kg IV every 24 h
Fluoroquinolones	
CIP	400 mg IV every 8–12 h or 500 mg PO every 8–12 h
LVX	750 mg every 24 h (IV or PO)
MOX	400 mg every 24 h (IV or PO)
*Metronidazole*	500 mg every 8-12 h or 1500 mg every 24 h (IV or PO)
Glycopeptides	
VAN	25–30 mg/kg IV loading dose, then 15–20 mg/kg IV every 8–12 h; target trough 15–20 mg/dL
TEC	12 mg/kg IV every 12 h for 3 doses (loading dose), then 6–12 mg/kg IV every 24 h
Oxazolidinone	
LZD	600 mg every 12 h (IV or PO)
Azoles	
FLC	800 mg IV loading dose then 400 mg IV every 24 h; 2 h infusion time
Echinocandins	
CAS	70 mg IV loading dose first day, then 50 mg IV daily
MFG	100 mg daily
AFG	200 mg loading dose first day, then 100 IV mg daily

*KEY: AFG* anidulafungin, *AMC* amoxicillin/clavulanic acid, *AMK* amikacin, *CAS* caspofungin, *CAZ* ceftazidime, *CIP* ciprofloxacin, *CRO* ceftriaxone, *CTX* cefotaxime, *CXM* cefuroxime, *CZA* ceftazidime/avibactam, *C/T* ceftolozane/tazobactam, *ETP* ertapenem, *FEP* cefepime, *FLC* fluconazole, *GEN* gentamicin, *IPM* imipenem, *IU* international units, *IV* intravenous, *LVX* levofloxacin, *LZD* linezolid, *MEM* meropenem, *MFG* micafungin, *MOX* moxifloxacin, *PO* per os, *TEC* teicoplanin, *TGC* tigecycline, *TZP* piperacillin/tazobactam, *VAN* vancomycin, *ZOX* ceftizoxime

### Duration of antimicrobial therapy in cIAI

The duration of antimicrobial therapy depends on (Tables 2, 3, 4, 5):

- Location of IAI (extrabiliary, biliary, and pancreatitis),
- Severity of illness,
- Adequacy of source control,
- Whether the infection is community- or hospital- acquired,
- Whether MDRO/XDRO are among the causative organisms,
- Clinical response (resolution of fever and leukocytosis, normalization or progressive improvement of the abdominal exam and of gastrointestinal function)

## Discussion

Antimicrobial resistance poses a global challenge, which calls for a global response; no area of the world is exempt from this pandemic [10, 48]. In general, the rational use of antimicrobials represents an integral part of good clinical practice [10]. The appropriateness of antimicrobial therapy is mostly dependent on the availability of regional epidemiological data and resistance profiles [10, 48]. The latter affects therapeutic efficacy of antimicrobials and minimizes the risks associated with the selection of resistant organisms [10]. The management of IAI is no exception to this issue, where the knowledge of regional/local rates of resistance, when available, is an essential component of the clinical decision-making process when concocting the empirical treatment of an infection [3, 19, 48]. In addition, strains of some resistant bacteria are endemic in certain geographic locations or may be limited to individual institutions or even to a specific unit within the same institution [49]. Accordingly, monitoring and updating community-, hospital- or unit-specific antibiograms are integral to providing effective therapy in a timely fashion in both the community and hospital settings [3, 19, 48].

In the context of antibiotic resistance surveillance and containment in IAI, the SMART project has provided comprehensive data about antibiotic resistance in IAI worldwide. Established in 2002, this surveillance system has monitored the in vitro antibiotic susceptibility patterns of clinical gram-negative bacilli collected worldwide from IAI samples [50]. Locally, the SMART surveillance report included combined data from Lebanon and Jordan [25]. Among 527 pathogens associated with IAI from 2011 through 2013, *E. coli*, *K. pneumoniae*, and *P. aeruginosa* were the most frequent species representing 46, 14, and 12% of the isolates, respectively [25]. The percentage of 3GC-resistance in *E. coli* and *K. pneumoniae*-related infections was 49 and 56%, respectively [25]. In 3GC-resistant *E.coli*, fluoroquinolone susceptibility ranged between 26 and 29%, with 97% susceptibility to imipenem [25]. In 3GC-resistant *K. penumoniae*, fluoroquinolone susceptibility ranged between 26 and 60%, and susceptibility to imipenem was 88% [25]. *A. baumannii* and *P. aeruginosa* isolates consistently showed low susceptibility patterns to tested antibiotics ranging from 4 to 8% and 75 to 89%, respectively [25]. The tested antibiotics included 3GC, fourth generation cephalosporins, piperacillin/tazobactam, fluoroquinolones, amikacin and imipenem [25].

In these guidelines, if we rely solely on SMART 3GC-resistance data, ranging between 50 and 60% in *E. coli* and *Klebsiellae spp*., as a crude microbiological platform for the empirical therapy of cIAI, we would end with overuse of carbapenems and/or tigecycline. The heavy use of carbapenems is a well-established predisposing factor for the colonization and infection with carbapenem-resistant Gram-negative pathogens. The rapid spread of carbapenem resistance in Enterobacteriaceae and in other non-lactose fermenting Gram-negative organisms in hospitals has jeopardized their therapeutic efficacy [48, 49]. Stewardship interventions pertaining to carbapenem sparing strategies along with its judicious use are integral to preserving the activity of this class of antimicrobials [48, 49]. In addition, the Lebanese SMART data represented pooled IAI data and were not stratified as community- or hospital-acquired as mentioned previously [25]. So to avoid the potential overuse of carbapenems, we classified patients into clinically stable and clinically unstable and whether the infection is community-acquired or nosocomial.

Owing to the concerns of increasing resistance to fluoroquinolones in different surveillance studies, ciprofloxacin and levofloxacin are no longer appropriate choice as first-line treatment in many geographic localities with a high prevalence of fluoroquinolone-resistant pathogens [25, 50]. They are prescribed prudently and restricted only to regions with 90% susceptibility rates [1]. Even in recently updated guidelines, a non-fluoroquinolone-based regimen would be preferable [3, 19].

Several studies in Lebanon have shown that patients infected with 3GC-resistant organisms in the community are at risk of carrying such resistant pathogens [26, 30, 51]. Reported risk factors were previous hospitalization, previous antibiotic intake, residing in a nursing home, etc. [26, 30, 51]. Accordingly, clinically stable patients with CA-cIAI with no such risk factors are managed with cephalosporins, while carbapenems should be restricted to clinically unstable patients or those at risk of contracting 3GC-resistant infections.

A recent compilation of antibiotic susceptibility data of bacteria isolated from different types of clinical samples from 13 Lebanese hospital laboratories during 2015 and 2016 showed that 40% of the Enterobacteriaceae were resistant to 3GCs and that carbapenem resistance in Enterobacteriaceae is emerging in these hospitals reaching an average of 3% (unpublished data). In Lebanon, the use of carbapenems in hospital setting is associated with a significant increase in the prevalence of carbapenem-resistant organisms including Enterobacteriaceae [52], *P. aeruginosa* [35], and *A. baumannii* [36]. Accordingly, in hospitals where resistance to 3GC is prevalent in Enterobacteriaceae and resistance to carbapenems in the same organisms is not yet endemic**,** we suggest using carbapenems in critically ill patients only [3]. However, in clinically stable patients with HA-cIAI, we suggest a carbapenem-sparing strategy. In this case, we recommend using an antipseudomonal beta-lactam combined with tigecycline. Tigecycline is a viable non-carbapenem option in empiric therapy due to its favorable in vitro activity against a large spectrum of resistant pathogens, including 3GC-resistant Enterobacteriaceae, CRE, *Acinetobacter* spp., and *Stenotrophomonas maltophilia* [53–55]. Additionally, it is approved by several international treatment guidelines [3, 19]. Similarly, polymyxins play a major role in combination therapy in critically ill patients infected with XDR pathogens [3, 19].

Ceftolozane/tazobactam and ceftazidime/avibactam are new antibiotics that have been approved for treatment of cIAI (in combination with metronidazole) caused by 3GC-resistant Enterobacteriaceae and *P. aeruginosa* [56–58]. Ceftolozane/tazobactam has been introduced to the Lebanese market in 2017. It exhibits a strong in vitro activity against XDR *P. aeruginosa*, showing stability against this bacterium’s common resistance mechanisms [59, 60]. Ceftazidime/avibactam appears to have in vitro activity against CRE [58]. Both antimicrobials would be valuable carbapenem-sparing options for treating infections caused by MDRO and XDRO [60]. In a recent systematic review and meta-analysis of randomized controlled trials comparing ceftolozane/tazobactam and ceftazidime/avibactam with other antibiotics for the treatment of cIAI, Chen et al. demonstrated that both agents were non-inferior to other available, well-established antimicrobial regimens for the treatment of cIAI in terms of clinical and microbiological success [61].

Shortening the duration of antimicrobial therapy, whenever possible, is a key measure in antimicrobial stewardship. The optimal duration of antibiotic therapy in case of cIAI has been a subject of debate. In patients with cIAI undergoing an adequate source-control procedure, a short course of antibiotic therapy (3–5 days) is recommended by recently updated guidelines [3, 19]. This recommendation is supported by several new studies [62–64]. A recent prospective trial by Sawyer et al. demonstrated that in patients with cIAI undergoing an adequate source control procedure, patient outcomes after 4 days of antibiotic therapy were similar to those after a longer course of antibiotics that extended until after the resolution of physiological abnormalities [62]. In critically ill patients with postoperative IAI, a recent multicenter prospective randomized trial conducted in 21 French intensive care units (ICU) between 2011 and 2015 compared the efficacy and safety of 8-day versus 15-day antibiotic therapy [63]. This trial was in favor of a short course of therapy and equivalence was established in terms of 45-day mortality [63]. Both arms did not differ in terms of ICU and hospital length of stay, emergence of MDR bacteria or reoperation rate [63]. Continuation of treatment until day 15 was not associated with any clinical benefit [63]. Interestingly, an excessive duration of antibiotic therapy for cIAI (8 days and above) was associated with subsequent extra-abdominal infection and significantly increased mortality, according to a single center study of 2552 consecutive IAI episodes from 1997 to 2010 in the United States [64].

A limitation of these guidelines resides in the absence of specific microbiological data about community- or hospital-acquired cIAI. Subsequently, they were based on extrapolating results from published literature about the resistance trends in Enterobacteriaceae from the Lebanese community and hospitals in general. Yet, these guidelines represent a major step towards initiating a Lebanese national antimicrobial stewardship program. Stratifying infected patients according to their clinical condition and site of infection could help overcome the uncertainty about the real prevalence of community-acquired bacterial resistance. Our approach can be used as a model for other resource-limited countries, where national surveillance of antibiotic resistance is not available, in order to break the vicious cycle of antibiotic overuse and emergence of resistance. Nevertheless, the LSIDCM emphasizes on development of a national AMR surveillance network that differentiates community-acquired from hospital-acquired IAI. A national antibiogram for cIAI should be established and stratified based on the setting (community, hospital, unit-based) and should be frequently updated. Accordingly, these guidelines are to be revised periodically.

## Conclusion

The antimicrobial therapy of cIAI is dependent upon the epidemiologic setting, severity of illness based on clinical assessment, local antibiotic susceptibility patterns and presence of risk factors for acquisition of resistant organisms. We recommend using non-3GC-containing antibiotic regimens for community-acquired infections when the following risk factors exist: prior (within 90 days) exposure to antibiotics, immunocompromised state, recent history of hospitalization or of surgery and invasive procedure all within the preceding 90 days. We also recommend initiating narrow spectrum antimicrobials then broadening the coverage in clinically stable patients and following an antimicrobial de-escalation strategy in critically ill patients. Prompt and adequate antimicrobial therapy for cIAI reduces morbidity and mortality; however, the duration of therapy should be limited to no more than 4 days when adequate source control is achieved. The management of acute pancreatitis is conservative, with a role for antibiotic therapy only in specific situations and after microbiological diagnosis. The use of broad-spectrum antimicrobial agents including systemic antifungals and newly approved antibiotics is preferably restricted to infectious diseases specialists. In Lebanon, the endemicity of 3GC-resistant Enterobacteriaceae in hospitals and the emergence of other carbapenem resistant gram-negative organisms emphasize the urgent need for the development and implementation of locally customized antibiotic stewardship programs, in addition to a rigorous antimicrobial resistance surveillance system.

## Abbreviations

3GC: 3rd generation cephalosporins
AP: Acute pancreatitis
APACHE: Acute Physiology and Chronic Health Evaluation
CA-cIAI: Community-acquired complicated Intra-abdominal Infections
cIAI: Complicated Intra-abdominal Infections
CRE: carbapenem-resistant Enterobacteriaceae
CRP: C-reactive protein
ERCP: Endoscopic retrograde cholangiopancreatography
ES: Endoscopic sphincterotomy
FNA: Fine needle aspiration
HA-cIAI: Hospital/Health care-associated complicated Intra-abdominal Infections
IAI: Intra-abdominal infections
ICU: Intensive Care Unit(s)
LSIDCM: Lebanese Society of Infectious Diseases and Clinical Microbiology
MDR: Multi-drug resistant
MDRO: Multi-drug resistant organisms
PCT: Procalcitonin
SMART: Study for Monitoring Antimicrobial Resistance Trends
XDR: Extensively-drug resistant
XDRO: Extensively-drug resistant organisms
